# Research on SVR Water Quality Prediction Model Based on Improved Sparrow Search Algorithm

**DOI:** 10.1155/2022/7327072

**Published:** 2022-04-28

**Authors:** Xuehua Su, Xiaolong He, Gang Zhang, Yuehua Chen, Keyu Li

**Affiliations:** School of Maritime and Transportation, Ningbo University, Ningbo 315211, China

## Abstract

Multiparameter water quality trend prediction technique is one of the important tools for water environment management and regulation. This study proposes a new water quality prediction model with better prediction performance, which is combined with improved sparrow search algorithm (ISSA) and support vector regression (SVR) machine. For the problems of low population diversity and easily falling into local optimum of sparrow search algorithm (SSA), ISSA is proposed to increase the initial population diversity by introducing Skew-Tent mapping and to help the algorithm jump out of local optimum by using the adaptive elimination mechanism. The optimal values of the penalty factor C and kernel function parameter *g* of the SVR model are selected using ISSA to make the model have better prediction accuracy and generalization performance. The performance of the ISSA-SVR water quality prediction model is compared with BP neural network, SVR model, and other hybrid models by conducting water quality prediction experiments with actual breeding-water quality data. The experimental results showed that the prediction accuracy of the ISSA-SVR model was significantly higher than that of other models, reaching 99.2%; the mean square deviation (MSE) was 0.013, which was 79.37% lower than that of the SVR model and 75% lower than that of SSA-SVR model, and the coefficient of determination (*R*^2^) was 0.98, which was 5.38% higher than that of the SVR model and 7.57% higher than that of the SSA-SVR model, indicating that the ISSA-SVR water quality prediction model has some engineering application value in the field of water body management.

## 1. Introduction

High-grade water environment quality is a necessary condition for improving the living environment and humanistic connotation of urban and rural residents, and with the increasing severity of global pollution, improving water environment quality is receiving much attention [1]. Water quality prediction technology uses the existing measured data to establish a water quality model to predict the future water environment conditions, which helps to understand the change pattern and development of the water environment and can provide technical support for water environment management and water pollution prevention [2]. In this case, the establishment of a suitable water quality prediction model to predict and assess water quality categories is a vital hydroenvironmental issue [3].

Traditional research on water quality prediction models has focused on linear methods [4], such as autoregression [5], autoregressive integrated moving average (ARIMA) [6] and other models in statistics [7], and neuron models [8]. However, the actual water quality factors are complex, diverse, and nonlinear, and traditional water quality prediction models cannot easily obtain the desired results for nonlinear data fusion problems [9].

In the past few decades, artificial intelligence (AI) in the form of machine learning models has been increasingly applied to solve various environmental engineering problems [10, 11], including river water quality modeling [3, 12]. AI models can fit nonlinear data well without detailed physical information, resulting in more accurate prediction results [13, 14]. Artificial neural networks (ANNs) [15], fuzzy logic-based models [16], support vector machines (SVMs) [17], and support vector regression (SVR) machines [18] have been widely used to predict and assess water quality.

Despite such broad usage of ANN models and fuzzy logic-based models, the models provided unsatisfactory results in some engineering problems. In previous studies, the combined form of AI models, known as hybrid AI models, has been extensively employed to solve such problems. Li et al. [19] proposed a novel model combining particle swarm optimization (PSO), chaos theory, self-adaptive strategy, and backpropagation artificial neural network (BP-ANN) to evaluate water quality. They found that the hybrid model is more effective than the BP-ANN. Penghui et al. [20] proposed a model (ANFIS-mSG) that hybridizes an adaptive neuro-fuzzy inference system with an optimization method using mutation salp swarm algorithm and grasshopper optimization algorithm. The modeling results evidenced the capability of optimization algorithms for building ANFIS models for simulating soil temperature.

In addition to hybrid fuzzy logic and ANN models, SVM have demonstrated an excellent predictive model for diverse engineering. SVR is an important application branch of support vector machine. The SVR method structure is more straightforward than fuzzy and ANN models that enhance the predicting model [21–23]. Therefore, the SVR method has more advantages in solving small samples, is nonlinear, and uses high-dimensional pattern recognition [24, 25]. These advantages of SVR make this method a popular choice for water quality prediction.

Although the SVR application has various advantages, it has some unknown parameters in its structure, which drastically affect the prediction accuracy and generalization performance. Therefore, optimization of the model parameters is required to obtain a prediction model with good performance. Many scholars have used metaheuristic algorithms for model parameter optimization due to their simple structure, fast efficiency, and few adjustment parameters [26, 27]. Doroudi et al. [28] used the observer-teacher-learner-based optimization (OTLBO) method to optimize the model parameters of the SVR model. The results indicate that the SVR-OTLBO model offers a higher prediction performance than other models employed in the current study. Haghbin et al. [29] used SVR combined with invasive weed optimization (IWO), standalone SVR, and Radial Basis Function neural networks to estimate channel sinuosity in perennial rivers. The results indicate that the sinuosity set predicted by the SVR-IWO model is the closest to the observed set. Tang et al. [30] optimized the model parameters based on multiagent particle swarm algorithm and proposed a new MAPSO-SVR predictive algorithm for the control prediction model of nonlinear systems with good performance.

Sparrow search algorithm (SSA) is a new metaheuristic algorithm proposed by Xue and Shen [31] in 2020. Sparrow search algorithm, particle swarm optimization (PSO) [32], and grey wolf optimizer (GWO) [33] are all swarm intelligent algorithms, which build models by simulating biological behavior. Sparrow search algorithm has been a wide concern because of its high efficiency, high convergence accuracy, and strong stability. Liu [34] established a prediction model based on sparrow search algorithm-optimized support vector machine regression (SSA-SVR) to predict the settlement of coal gangue roadbed of An Shao Expressway in Hunan Province and compared the prediction results with PSO-SVR and GA-SVR models. The results show that the SSA-SVR prediction model has high accuracy and good generalization ability. Xu et al. [35] proposed to use the sparrow search algorithm (SSA)-optimized SVR model for training to obtain a soft measurement model capable of identifying the dynamic viscosity of the target fluid. The results show that the SSA-SVR soft measurement model-based liquid viscosity identification method can quickly and effectively identify the dynamic viscosity of liquids and the detection accuracy in the studied viscosity range is better than that of traditional measurement methods.

However, SSA, like other population intelligence optimization algorithms, still suffers from the problems of decreasing population diversity and easily falling into local optimality when its search is close to the global optimum [31]. In order to overcome the shortcomings of traditional SSA, many scholars have improved SSA by increasing the initial population diversity and escaping from the local optimum. Lv et al. [36] use Tent chaotic mapping to initialize the population so that the initial individuals are distributed as evenly as possible, while introducing Gaussian variation and chaotic perturbation to help individuals jump out of the local optimum, thus overcoming the drawback that SSA is prone to fall into the local optimum. Mao et al. [37] integrated the idea of sine-cosine algorithm to balance the local and global searchability in the discoverer position update method of SSA and introduced Levy flight strategy in the follower position update method to perturb the variation of the current optimal solution and strengthen the local escape ability, which obviously improved the efficiency of SSA solution. Tang et al. [38] used cubic mapping to initialize the population to obtain an improved chaotic sparrow search algorithm, while using the Gaussian wandering strategy to help the algorithm jump out of stagnation. Simulation results show that the algorithm outperforms the particle swarm algorithm (PSO), the beetle swarm optimization algorithm (BSO), the whale optimization algorithm (WOA), the grey wolf optimization algorithm (GWO), and the sparrow search algorithm (SSA) in finding the optimal performance.

The improvement of SSA in the above literature can avoid the algorithm from falling into local optimum and improve the searchability to a certain extent, but there are still defects such as insufficient search accuracy and weak pioneering ability of the algorithm. For the improved population intelligence optimization algorithm, the chaotic variables generated by Skew-Tent mapping have better traversal uniformity, which can effectively shorten the search time and better reduce the sensitivity dependence of initial values. Li and Xu [39] used Skew-Tent mapping to permute the pixels in an image, which greatly improved the encryption performance and security of digital image encryption algorithm. Meng et al. [40] proposed a new image encryption algorithm using Skew-Tent mapping for pixel permutation to improve the security of the system, and the results showed that the algorithm outperformed several advanced image encryption algorithms. For adding an optimization strategy to help the algorithm jump out of the local optimum, the adaptive search strategy dynamically adjusts the number of elements in the candidate set for the centralized search and the diversity search, respectively, through the cooperation of the neighborhood and the candidate set, which better solves the conflict problem of centralization and diversity. Kong et al. [41] introduced the adaptive adjustment weight method and search strategy to improve the ability of the WOA algorithm to jump out of the local optimum. Liu et al. [42] introduced an adaptive boundary mechanism in the process of ants wandering around the ant-lion to increase the ant population activity and prevent the basic ant-lion optimization algorithm (ALO) from falling into local extremes. The test results show that the proposed algorithm has significantly improved optimization-seeking accuracy and convergence speed, is little affected by dimensional changes, and has stronger and more stable high-dimensional solving ability.

Considering the traversal uniformity and fast convergence of the Skew-Tent mapping and the better local searchability of the adaptive search strategy, this study proposes an improved sparrow search algorithm (ISSA). ISSA first introduces the Skew-Tent mapping to initialize the population to increase the population diversity and then uses the adaptive elimination mechanism to improve the population position update strategy of the algorithm, which improves the global searchability of the algorithm. Then, the ISSA-SVR model was established by combining the ISSA with the support vector regression machine, which is to optimize the penalty factor C and kernel function parameter *g* of support vector regression using ISSA, so as to obtain a water quality prediction model with better prediction accuracy and generalization performance. Finally, water quality category prediction experiments were conducted with actual aquaculture water quality data to verify the reliability and stability of the model.

## 2. Improved Sparrow Search Algorithm

Although SSA has the advantages of fast convergence, high stability, few adjustment parameters, and simple computation, it also has the disadvantage of easily falling into the local optimum, just like other intelligent search algorithms. For this reason, this paper makes improvements to SSA.

### 2.1. Standard Sparrow Search Algorithm

Sparrow search algorithm (SSA) is a novel intelligent search algorithm that simulates sparrow foraging and antipredation behaviors, proposed by Xue and Shen [31] in 2020. Sparrows are flock birds in nature, with a clear division of labor within the population. There are three behavioral groups of sparrows in the foraging process: the discoverer searches for food; the joiner follows the discoverer to find food; and the warner alerts to avoid danger. The roles of the discoverer and the joiner are dynamically interchanged, but the proportion of the discoverer and the joiner in the population remains the same and the warner is randomly generated in the population.

Assuming that the entire population of sparrows is *N*, the position of the *i*-th sparrow in the D-dimensional search space is *X*_*i*_=[*x*_*i*1_, *x*_*i*2_,…, *x*_*i*  *D*_],  *i*=1,2,…, *N*.

Generally, the discoverer accounts for 10% to 20% of the total population, and the position update formula of the discoverer in each iteration is as follows:

(1) $$\begin{array}{c} X_{i}^{t+1}=\left\{\begin{array}{cc} X_{i}^{t}\cdot \;\exp\left(-\frac{i}{\alpha \cdot T}\right), & R_{2}<\text{ST},\\ X_{i}^{t}+Q\cdot L, & R_{2}\geq \text{ST,} \end{array}\right. \end{array}$$

where *α* ∈ (0,1) is a random number; *T* is the maximum number of iterations; *t* is the current number of iterations; *X*_*i*_^*t*^ is the position information of the *i*-th sparrow at the *t*-th iteration; *Q* is a random number that obeys a normal distribution; *L* is a 1 × *D*-dimensional matrix whose elements are all 1, ST ∈ [0.5, 1] is the warning value; ST ∈ [0.5, 1] is the safety value; and *R*_2_ ∈ [0,1] is the warning value. When *R*_2_ < ST, it means that the environment is safe and the discoverer can search extensively at this time; when *R*_2_ ≥ ST, it means that some individuals in the population find environmental dangers and give an early warning; at this time, all individuals fly to the safe area to search.

All remaining in the population except the discoverer are the joiner. The formula for updating the position of the joiner is as follows:

(2) $$\begin{array}{c} X_{i}^{t+1}=\left\{\begin{array}{c} Q\cdot \;\exp\left(\frac{X_{\text{worst}}-X_{i}^{t}}{i^{2}}\right),\;i>\frac{n}{2},\\ X_{p}^{t+1}+\left|X_{i}^{t}-X_{p}^{t+1}\right|\cdot A^{+}\cdot L,\;i\leq \frac{n}{2}, \end{array}\right. \end{array}$$

where *X*_worst_ is the current global worst position; *X*_*p*_ is the current optimal position of the discoverer; and *A*^+^=*A*^*T*^(AA^*T*^)^−1^, where A is a 1 × *D*-dimensional matrix with each element randomly assigned a value of 1 or −1. When *i* > *n*/2, the *i*-th individual with lower fitness has a poor search position and needs to fly to other places to search.

The warner in the sparrow population generally accounts for 10% to 20% of the total population, and the initial position of the warner is randomly generated at the initial stage of the population formation. The formula for updating the position of the warner is as follows:

(3) $$\begin{array}{c} X_{i}^{t+1}=\left\{\begin{array}{c} X_{\text{best}}^{t}+\beta \cdot \left|X_{i}^{t}-X_{\text{best}}^{t}\right|,\;f_{i}\neq f_{g},\\ X_{i}^{t}+K\cdot \left(\frac{\left|X_{i}^{t}-X_{\text{worst}}^{t}\right|}{\left(f_{i}-f_{w}\right)+\varepsilon }\right),\;f_{i}=f_{g}, \end{array}\right. \end{array}$$

where *X*_best_ is the current global optimal position; *β* is a random number that obeys a normal distribution with a mean of 0 and a variance of 1, representing the step-length control parameter; *K* ∈ [−1,1] is a random number; *f*_*i*_ is the fitness value of the *i*-th individual; *f*_*g*_ is the current global optimal fitness value; *f*_*w*_ is the current global worst fitness value; and *ε* is the smallest constant. When *f*_*i*_ ≠ *f*_*g*_, it means that the *i*-th individual is at the edge of the population and is easily attacked; when *f*_*i*_=*f*_*g*_, it means that the individual in the center of the population is aware of the danger and needs to approach other individuals to avoid danger.

### 2.2. Skew-Tent Map

SSA usually uses random initialization of populations when solving optimal problems, which can easily cause uneven distribution of populations and lead to the reduction of population diversity [31]. It has been shown that the goodness of population initialization in the intelligent search algorithm affects the accuracy and convergence speed of the algorithm and the initial population with better diversity is also more helpful to the final performance improvement of the algorithm. In solving the global optimal problem, since there is no more a priori knowledge to draw from, increasing the population diversity as much as possible can improve the search efficiency of the algorithm and lay the foundation for the global search of the algorithm.

A chaotic phenomenon occurs in nonlinear systems, which is a deterministic, stochastic-like process, but the process is nonconvergent, nonperiodic, and bounded. Skew-Tent mapping [43] is a widely used and studied chaotic mapping formula due to its simple structure and iterative process that removes rounding errors. In this paper, the Skew-Tent mapping is used to generate chaotic variables with ergodicity, randomness, and regularity to initialize the population of the SSA to produce an initial population with high diversity. Assuming that the number of populations is *N* and the optimization problem is *D*-dimensional, a chaotic sequence matrix is generated in the *D*-dimensional Euclidean space using the Skew-Tent mapping:

(4) $$\begin{array}{c} Y=\left[\begin{array}{cccc} y_{1,1} & y_{1,2} & \cdots & y_{1,D}\\ y_{2,1} & y_{2,2} & \cdots & y_{2,D}\\ \vdots & \vdots & \vdots & \vdots \\ y_{N,1} & y_{N,2} & \cdots & y_{N,D} \end{array}\right]. \end{array}$$

The Skew-Tent map expression is as follows:

(5) $$\begin{array}{c} y_{i+1,d}=\left\{\begin{array}{cc} \frac{y_{i,d}}{a}, & y_{i,d}\in \left(0,a\right),\\ \frac{y_{i,d}-1}{a-1}, & y_{i,d}\in \left(a,1\right), \end{array}\right. \end{array}$$

where *a* ∈ (0,1) is a random number, *i*=1,2,…, *N*, and *d*=1,2,…, *D*.

The sparrow population is a *N* × *D*-dimensional matrix. This paper uses Skew-Tent map to generate the chaotic series matrix to initialize the sparrow population. The formula is as follows:

(6) $$\begin{array}{c} x_{i,d}=x_{i,d\_\min}+\left(x_{i,d\_\max}-x_{i,d\_\min}\right)\times y_{i,d}, \end{array}$$

where *x*_*i*,*d*_min_ and *x*_*i*,*d*_max_ are the upper and lower limits of the *i*-th individual on the *d*-th dimension and *x*_*i*,*d*_ is the actual value of the *i*-th individual on the *d*-th dimension.

### 2.3. Adaptive Elimination Mechanism

SSA is prone to fall into local optimal solutions when the population search is close to the optimal solution. In order to improve the global searchability and convergence speed of SSA, this paper introduces an adaptive elimination mechanism. Assuming that the number of populations is *N* and the maximum number of iterations of SSA is *T*, an elimination rate *ω* is introduced, which is calculated as follows:

(7) $$\begin{array}{c} \omega =\left\{\begin{array}{cc} 0.5, & 0.3<\frac{t}{T},\\ 0.3, & 0.3\leq \frac{t}{T}<0.6,\\ 0.2, & 0.6\leq \frac{t}{T}<0.8,\\ 0.1, & 0.8\leq \frac{t}{T}, \end{array}\right. \end{array}$$

where *t* is the current iteration number.

At each iteration, the fitness of the entire population is calculated and *l*=*N* · *ω* individuals are eliminated in the descending order of fitness. To ensure that the population size remains constant, new individuals must be added to the population. To improve the utilization of prior knowledge, the location information of eliminated individuals is updated by randomly selecting individuals from the current number of iterations, and the formula for updating the location of new population individuals is as follows:

(8) $$\begin{array}{c} X_{i}^{t\prime }=X_{i}^{t}+\varepsilon \cdot \left(X_{j}^{t}-X_{k}^{t}\right), \end{array}$$

where *ε* ∈ (0,1) is the scaling factor; *X*_*j*_^*t*^ and *X*_*k*_^*t*^ represent the positions of two randomly selected individuals in the *t*-th iteration; and *X*_*i*_^*t*′^ and *X*_*i*_^*t*^ are the new and old position information of the currently eliminated individual, respectively.

### 2.4. Implementation

The pseudo-code of the improved sparrow search algorithm is expressed in Algorithm 1.

### 2.5. Taguchi-Grey Relation Method

Obtaining the optimum parameters of the optimization algorithms is a major concern in the current study [44]. The Taguchi method [45] aims to select the optimal combination of parameters by calculating the signal-to-noise (SN) ratio through orthogonal tests, which has the advantages of fewer tests, reliable test results, good reproducibility, and simple analysis and calculation, and has been widely used. However, the traditional Taguchi method can only solve single-objective optimization problems at a time, while the problems we encounter in practice mostly require simultaneous consideration of multiple objectives. The Taguchi-grey relation method combines Taguchi method and grey relational analysis [46] to effectively solve multiobjective design optimization problems. Therefore, the Taguchi-grey relation method is used to obtain the parameter combination of ISSA with the minimum number of iterations and the best robustness.

#### 2.5.1. Orthogonal Experiment

According to the implementation of ISSA, three control factors that have three levels each (see Table 1) were selected as the control factors of the Taguchi method. Since the experiment had three control factors with three levels per factor, the experimental protocol was set at 9 according to the general principles of orthogonal table design and experience, so the orthogonal array can be expressed as *L*_9_(3^3^); the specific experimental protocol is shown in Table 2.

According to the basic evaluation index of the optimization algorithm [47, 48], the global convergence probability (GP), volatility, and average execution time are selected as the optimization objectives.

The global convergence probability is an optimization performance indicator, which reflects the global convergence performance of the algorithm and is denoted as GP:

(9) $$\begin{array}{c} \text{GP}=\frac{n}{N}, \end{array}$$

where *n* is the number of convergence times and *N* is the total number of runs.

Volatility is a robust indicator, which can measure how close the algorithm is to the optimal solution under random initial values, and is denoted as *V*:

(10) $$\begin{array}{c} V=\frac{f^{*}-\sum_{i=1}^{N}f_{i}/N}{f^{*}}, \end{array}$$

where *f*^*∗*^ is the optimal value of the objective function and ∑_*i*=1_^*N*^*f*_*i*_/*N* is the mean value of the objective function in *N* runs.

Average execution time is a time performance indicator, which can measure the speed of the algorithm's search for the solution of the problem, denoted as $\bar{t}$:

(11) $$\begin{array}{c} \bar{t}=\sum_{i=1}^{N}\frac{t_{i}}{N}, \end{array}$$

where *t*_*i*_ is the CPU execution time spent in each run of the algorithm.

By using SN ratio processing, the change trends of optimization objectives with different characteristics can converge to the same direction, all the larger the better, which is convenient for subsequent analysis and calculation.

Among them, the global convergence probability belongs to the bigger-the-better characteristic and its SN ratio is calculated as follows:

(12) $$\begin{array}{c} \frac{S}{N}=10\mathrm{lg}\left(\frac{1}{T}\sum_{i=1}^{T}y_{i}^{2}\right), \end{array}$$

where *y*_*i*_ is the value of the *i*-th experiment of the optimization objective and *T* is the number of tests under the same test parameters, and this paper takes *T*=1.

Volatility and average execution time belong to the smaller-the-better characteristic, and the formula for calculating the SN ratio is

(13) $$\begin{array}{c} \frac{S}{N}=-10\mathrm{lg}\left(\frac{1}{T}\sum_{i=1}^{T}y_{i}^{2}\right). \end{array}$$

According to the given orthogonal table, the signal-to-noise ratios of the three indicators are calculated, and the results are shown in Table 2.

**Figure 1:**
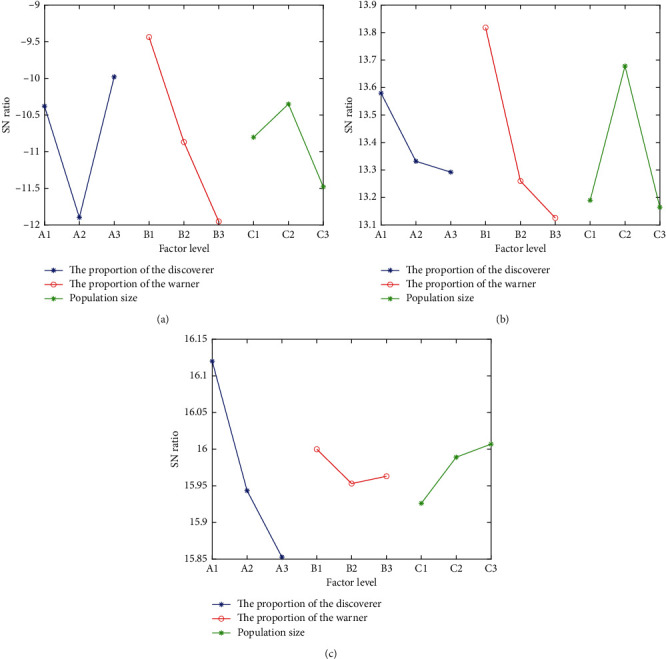
Response plots of average SN ratio. Response plot for (a) GP, (b) *V*, and (c) $\bar{t}$ at each factor and level.

The average SN ratio of the three optimization objectives at each factor and each level can be calculated based on the SN ratio in Table 2, and the results are shown in Tables 3–5 and Figure 1. The max-min indicated the level of most influencing nature among the control factors. Therefore, it can be seen from Tables 3–5 that population size has the greatest impact on GP, the proportion of the warner has the greatest impact on *V*, and the proportion of the discoverer has the greatest impact on $\bar{t}$. Figure 1 shows that the optimal parameter combinations for each optimization objective are different, i.e., the optimal parameter combination for GP is A3B1C2, the optimal parameter combination for *V* is A1B1C2, and the optimal parameter combination for *t* is A1B1C3, so the grey correlation analysis is performed subsequently to select a unique optimal parameter combination.

#### 2.5.2. Grey Relational Analysis

The grey relational analysis is used to solve the interrelationship among multiple responses. This analysis includes the following steps.

**Step 1 .**

The weight coefficients of the three performance evaluation indicators are determined according to AHP [49]. First, we establish the corresponding judgment matrix *A*=[1,7,9; 1/7, 1,3; 1/9, 1/3, 1] according to experience and then obtain the eigenvector AW=(0.776, 0.155,  0.069) and the eigenvalue *b*=3.083. After the consistency test, the judgment matrix *A* meets the consistency requirements. Therefore, the weight vector of the three performance evaluation indicators is *w*=(0.776,  0.155,  0.069).

**Step 2 .**

We normalize the S/N ratio to distribute the data evenly and scale it into acceptable range for further analysis by using equations. Then, the following formulas are used to calculate the grey relational coefficient (GC) of the three performance evaluation indicators:

(14) $$\begin{array}{c} {\text{GC}}_{i}\left(k\right)=\frac{\underset{i}{\min}\underset{k}{\min}\left|x_{0}\left(k\right)-x_{i}\left(k\right)\right|+\xi \underset{i}{\max}\underset{k}{\max}\left|x_{0}\left(k\right)-x_{i}\left(k\right)\right|}{\left|x_{0}\left(k\right)-x_{i}\left(k\right)\right|+\xi \underset{i}{\max}\underset{k}{\max}\left|x_{0}\left(k\right)-x_{i}\left(k\right)\right|}, \end{array}$$

where GC_*i*_(*k*) is the grey relational coefficient for the *k*-th performance characteristics in the *i*-th experiment, *X*_0_={*x*_0_(1), *X*_0_(2),…, *X*_0_(*n*)} is the reference sequence in the analysis, *X*_i_={*x*_*i*_(1), *X*_*i*_(2),…, *X*_*i*_(*n*)} is the sequence to be compared in the analysis, and *ξ* is the resolution coefficient; *ξ* ∈ [0,1], and usually, *ξ* is taken as 0.5.

**Step 3 .**

The grey relational degree is calculated according to the weight vector in Step 1, as shown in Table 6.

**Step 4 .**

**Figure 2:**
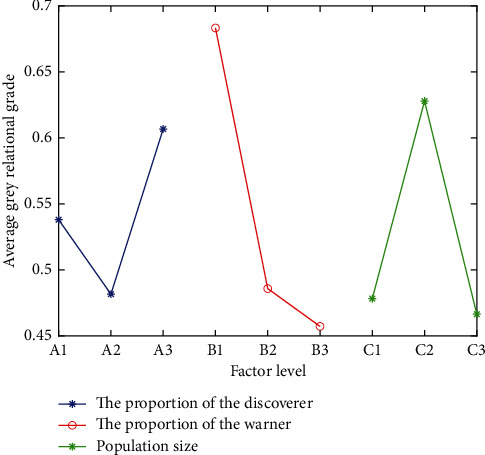
Response plot of the average grey relational grade.

We utilize the response graph method to select optimal levels of the control factors based on the maximum average grey relational grade.  Table 7 shows the average of grade scale for all the levels of control factors, and the proportion of the warner has the greatest impact on the grey relational grade.  Figure 2 shows the response graph of the average grey relational grade. It has been observed that the better optimal value of parameters is A3B1C2, which means the proportion of the discoverer is 20%, the proportion of the warner is 10%, and the population size is 30.

## 3. ISSA Simulation Experiments

The purpose of the simulation experiment is to validate the numerical efficiency of ISSA in comparison with other popular optimization algorithms. The experiment is to test ISSA with other optimization algorithms over a series of benchmark functions [50]. These benchmark functions provide patterns of search spaces of different optimization challenges, usually filled with many local optima.

### 3.1. Benchmark Functions

Unimodal functions have only one extreme point, which can be used to verify the algorithm's convergence speed, optimization accuracy, and local development ability. Multimodal functions have multiple local extremum points, which makes the algorithm extremely easy to fall into the local extremum and can be used to verify the algorithm's ability to escape from the local extremum and global exploration ability. Fixed-dimension multimodal functions can further verify the convergence speed, stability, and convergence accuracy of the algorithm. Therefore, seven unimodal functions (F1–F7), six multimodal functions (F8–F13), and four fixed-dimension multimodal functions (F14–F17) were selected for experiments in this paper. Tables 8–10 summarize the test problems reporting the cost function, the range of variation of optimization variables, and the optimal value quoted in the literature.

### 3.2. Parameter Settings

ISSA was compared with SSA, TLBO, SOA, GWO, PSO, and WOA, and the experimental parameter settings of each algorithm are shown in Table 11. Note that the experimental parameter settings of the comparative algorithms are taken from [32, 33, 51, 52]. For fairness, the population size of each algorithm is set to 30 and the maximum number of iterations is 500.

### 3.3. Results and Analysis

**Figure 3:**
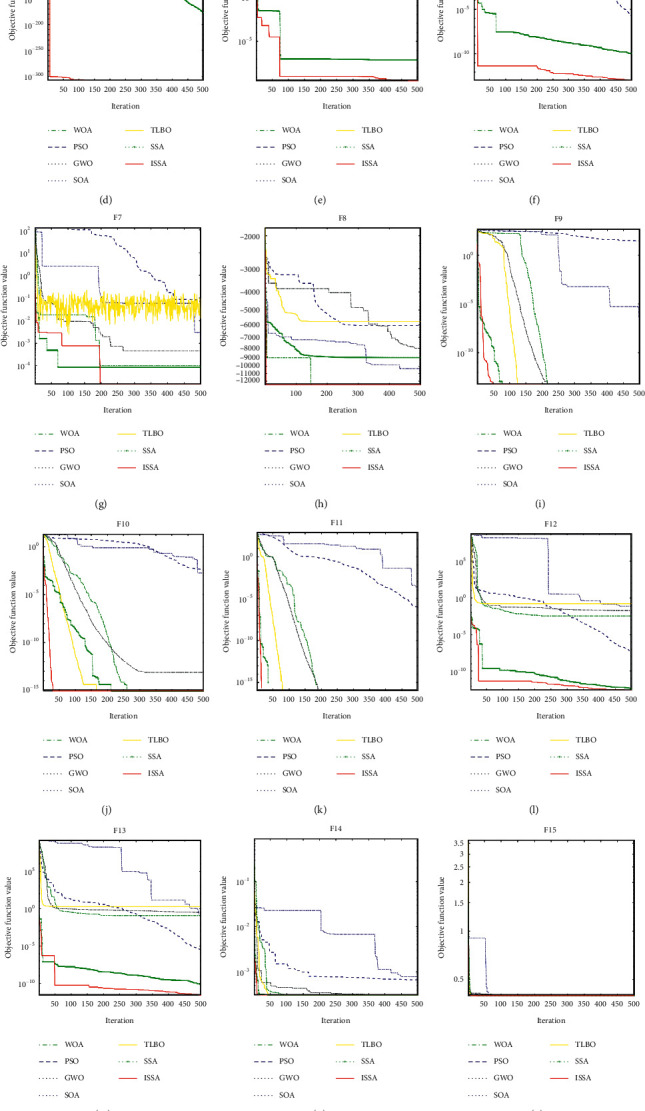
Convergence curve of benchmarking functions. (a) F1 iteration convergence curve. (b) F2 iteration convergence curve. (c) F3 iteration convergence curve. (d) F4 iteration convergence curve. (e) F5 iteration convergence curve. (f) F6 iteration convergence curve. (g) F7 iteration convergence curve. (h) F8 iteration convergence curve. (i) F9 iteration convergence curve. (j) F10 iteration convergence curve. (k) F11 iteration convergence curve. (l) F12 iteration convergence curve. (m) F13 iteration convergence curve. (n) F14 iteration convergence curve. (o) F15 iteration convergence curve. (p) F16 iteration convergence curve. (q) F17 iteration convergence curve.

The simulation experiments in this paper are carried out using MATLAB 2018b software. The parameter settings of the seven algorithms are shown in Table 11. The performance of the algorithm is verified using the test functions shown in Tables 8–10. To avoid the result bias caused by chance, the seven algorithms are run 30 times on each test function separately. Its optimal value, mean value, and standard deviation are recorded in Table 12. To better reflect the optimization effect of the algorithm, this paper provides the convergence curve of ISSA, SSA, TLBO, SOA, GWO, PSO, and WOA for the above sixteen test functions, as shown in Figure 3.

#### 3.3.1. Unimodal Functions (Functions F1–F7)

1. *Convergence Accuracy Analysis*. As shown in Table 12, ISSA finds the optimal values for F1, F3, and F4. Although the optimal values are not found for F2, F5, and F6, they are also significantly better than those of the other algorithms. For F7, both ISSA and SSA are significantly better than other algorithms, and ISSA is slightly better than SSA in terms of the average and optimal values obtained.
2. *Stability Analysis*. From the STD test data in Table 12, it can be seen that ISSA has significantly better standard deviation than other algorithms for F1, F3, and F6 and slightly better standard deviation than SSA for F2, F5, and F7. The ISSA is significantly better than other algorithms. In processing F4, ISSA is less stable than TLBO but better than other algorithms. In conclusion, the simulation experiments show that the sparrow search algorithm has certain stability in the unimodal test function and also has certain advantages compared with other three algorithms.
3. *Convergence Speed Analysis.* From Figures 3(a)–3(f), we can see that ISSA has absolute advantage in convergence speed on F1, F3, and F4 and converges to the optimal value faster than other algorithms on F2, F5, and F6. On F7, although ISSA converges slower than SSA, the convergence to the value is better than SSA.

In summary, we can conclude that our proposed ISSA can find the ideal value quickly and has strong optimization and exploitation capabilities when dealing with unimodal test functions.

#### 3.3.2. Multimodal Functions (Functions F8–F13)

1. *Convergence Accuracy Analysis.* As shown in Table 12, ISSA finds the optimum for F8, F9, and F11 and is the only algorithm that finds the optimum for F8. ISSA, SSA, and WOA have almost the same searchability on F10.
2. *Stability Analysis.* In the F8 test function, although ISSA outperforms the other algorithms in terms of solution accuracy, its stability is relatively poor. For the remaining test functions F9–F13, ISSA has better stability. In conclusion, from these results, we can clearly see that the ISSA has good stability and strong adaptability in dealing with multimodal test functions, also reflecting that the algorithm has the ability to mine the optimal solution.
3. *Convergence Speed Analysis.* From the convergence curves in Figures 3(g)–3(l), we can see that ISSA can converge to the optimal value within 50 iterations on F8–F11, which is obviously better than other algorithms. The convergence speed on F12 and F13 is also significantly better than the other algorithms, although it is only slightly better than SSA. From the figure, it can be seen that SSA has faster convergence speed and better ability to explore unknown regions when dealing with high-dimensional and complex problems.

In summary, by processing multimodal test functions, we further conclude that ISSA has a strong global search capability and is adaptable to a variety of different test functions. The Skew-Tent mapping makes a great contribution to the global search.

#### 3.3.3. Fixed-Dimension Multimodal Functions (Functions F14–F17)

1. *Convergence Accuracy Analysis*. Based on the analysis of the results in Table 12, it is concluded that for F15, all four algorithms are able to search for the optimal value quickly and efficiently. This result also proves that the optimization of these algorithms on this test function is very successful. For F16 and F17, ISSA, SSA, TLBO, GWO, and PSO can find the optimal values, but the analysis of the average value shows that ISSA outperforms the other algorithms. For F14, the optimal values of ISSA, SSA, and TLBO can be found and SSA is slightly better than ISSA according to the average value of the analysis.
2. *Stability Analysis.* From the standard deviations obtained in Table 12 for the optimization of F14, we can see that the standard deviations of TLBO, SOA, GWO, PSO, and WOA are larger, which means that the stability is relatively poor, while the standard deviation of ISSA is obviously much smaller, which means that the data distribution is more concentrated and the stability is better. For the F15 test function, the stability of ISSA, SSA, TLBO, and PSO is better than that of SOA, GWO, and WOA. For the F16 test function, the stability of ISSA, SSA, TLBO, GWO, and PSO is better and that of SOA and WOA is worse. For F17, the stability of ISSA is slightly better than that of other six algorithms.
3. *Convergence Speed Analysis.* The convergence curves are shown in Figures 3(m)–3(p), and these four fixed-dimension multimodal test functions have a strong convergence speed on F15. For F14, the convergence curve shows that the convergence speed of ISSA is faster than that of the other algorithms, which is because ISSA converges quickly to a stable value at the beginning of the iteration. For F17, the convergence trends of ISSA, SSA, TLBO, and WOA are consistent throughout the optimization process, the convergence curves of ISSA and SSA basically overlap, and the convergence rates are close. For F16, the convergence trends of ISSA, SSA, and TLBO are consistent throughout the optimization process and the convergence speed of TLBO is slightly faster than that of ISSA.

The simulation results show that ISSA has strong optimization ability for unimodal test function, multimodal test function, and fixed-dimension multimodal test function. In particular, ISSA has a more obvious competitive advantage than other algorithms in solving the multimodal test function. Through comparison, we find that ISSA can still give very competitive results compared with other state-of-the-art algorithms, and ISSA has improved accuracy, stability, and convergence speed compared with SSA. It can be concluded that ISSA has good robustness and convergence speed and is an effective tool for solving optimization problems.

## 4. ISSA-SVR Water Quality Regression Prediction Model

Based on the actual aquaculture water quality data, this paper uses the improved sparrow search algorithm to optimize the parameters of the SVR model, optimizes the penalty factor C and kernel function parameter *g*, so as to obtain the appropriate model parameters, and constructs the SVR water quality regression prediction model with the best prediction accuracy.

### 4.1. Support Vector Regression

Support vector regression (SVR) is an application of SVM in the field of regression prediction. SVR finds a linear regression equation, which can calculate a hyperplane to minimize the total variance of the sample points from the hyperplane to fit all sample points. SVR has usually been used to deal with nonlinear regression problems through a kernel function mapping low-dimensional sample space to high-dimensional feature space.

Let {(*x*_1_, *y*_1_),…, (*x*_*i*_, *y*_*i*_),…, (*x*_*m*_, *y*_*m*_)} be a training sample set with a given capacity of *m*, where *x*_*i*_=*R*^*n*^ is the input space with n-dimension and *y*_*i*_=*R* is the output space. For nonlinear problems, SVR fitting function can be expressed as

(15) $$\begin{array}{c} g\left(x\right)=w^{T}\cdot \phi \left(x\right)+b, \end{array}$$

where *ϕ*(*x*) is the mapping function, which can map low-dimensional nonlinear samples to high-dimensional feature space; *w* is the regression coefficient; and *b* ∈ *R* is the intercept term.

Considering that there may be some errors in solving practical problems, two slack variables *ξ*^(*∗*)^=(*ξ*_1_, *ξ*_1_^*∗*^,…,*ξ*_*m*_, *ξ*_*m*_^*∗*^)^*T*^ and  *i*=1,2,…, *m* are introduced within the allowable range of errors. Therefore, the regression fitting problem can be expressed as

(16) $$\begin{array}{c} \left\{\begin{array}{cc} \frac{1}{2}\min & {\left\|w\right\|}^{2}+C\sum_{i=1}^{m}\left(\xi_{i}+\xi_{i}^{*}\right)\\ \text{s.t.} & \begin{array}{c} g\left(x_{i}\right)-y_{i}\leq \varepsilon +\xi_{i},i=1,2,\ldots ,m\\ y_{i}-g\left(x_{i}\right)\leq \varepsilon +\xi_{i}^{*},i=1,2,\ldots ,m\\ \xi_{i}\geq 0,\xi_{i}^{\left(*\right)}\geq 0,i=1,2,\ldots ,m, \end{array} \end{array}\right. \end{array}$$

where *C* ≥ 0 is the penalty factor; *ε* is the deviation between the predicted value and the true value; and *ξ*_*i*_ ≥ 0 and *ξ*_*i*_^(*∗*)^ ≥ 0 are the slack variables of the upper and lower bounds, respectively.

By introducing the kernel function *k*(*x*_*i*_, *x*_*j*_)=*ϕ*(*x*_*i*_)^*T*^ · *ϕ*(*x*_*j*_), the input samples are mapped to the high-dimensional feature space, and according to the Lagrange duality, the nonlinear problem is converted to the following convex quadratic programming problem:

(17) $$\begin{array}{c} \left\{\begin{array}{cc} \underset{a^{\left(*\right)}\in R^{2m}}{\min} & \frac{1}{2}\sum_{i,j=0}^{m}\left(\alpha_{i}^{*}+\alpha_{i}\right)\left(\alpha_{i}^{*}+\alpha_{i}\right)k\left(x_{i},x_{j}\right)+\varepsilon \sum_{i=1}^{m}\left(\alpha_{i}^{*}+\alpha_{i}\right)-\sum_{i=1}^{m}y_{i}\left(\alpha_{i}^{*}-\alpha_{i}\right)\\ \text{s.t.} & \sum_{i=1}^{m}\left(\alpha_{i}^{*}-\alpha_{i}\right)=0,\;0\leq \alpha_{i},\alpha_{i}^{*}\leq C,i=1,2,\ldots ,m, \end{array}\right. \end{array}$$

where *α*_*i*_, *α*_*i*_^*∗*^ ≠ 0 are the Lagrange multipliers.

According to KKT condition, the decision function of the SVR model under the nonlinear problem can be obtained after the final solution is

(18) $$\begin{array}{c} f\left(x\right)=\sum_{i=1}^{m}\left(a_{i}^{*}-a_{i}\right)K\left(x_{i}\cdot x\right)+b. \end{array}$$

This paper uses Gaussian Radial Basis Function (RBF), and the expression is as follows:

(19) $$\begin{array}{c} k\left(x,x_{i}\right)=\exp\left(\frac{-{\left\|x-x_{i}\right\|}^{2}}{2g^{2}}\right), \end{array}$$

where *g* > 0 is the bandwidth of the Gaussian kernel and the kernel function parameter. From the above derivation, it can be seen that the key parameters affecting SVR are the penalty factor C and the kernel function parameter *g*.

### 4.2. ISSA-SVR Water Quality Prediction Model

**Figure 4:**
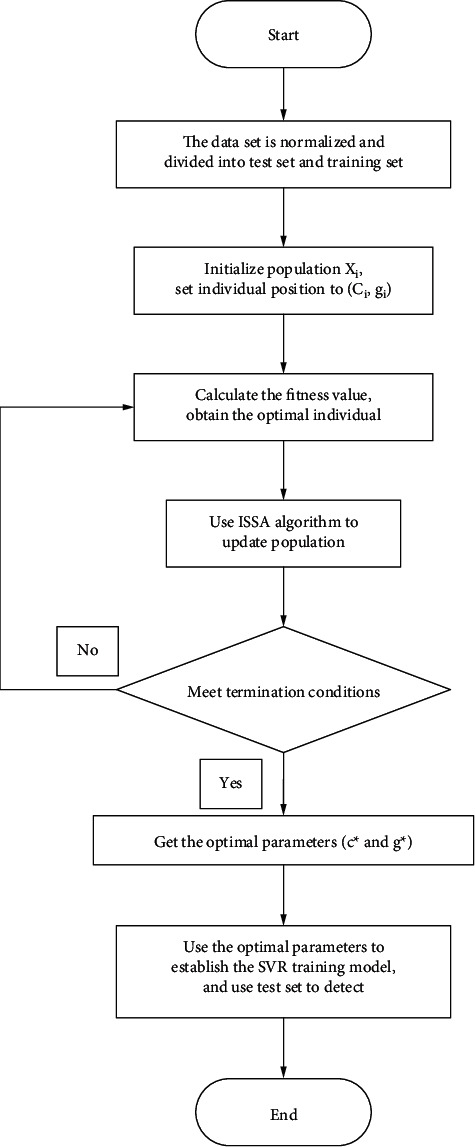
Flow chart of the ISSA-SVR prediction model.

The performance of the SVR prediction model is mainly determined by the penalty factor C and the kernel function broadband *g*. Blind parameter selection is likely to cause the problem of low accuracy and low efficiency of the SVR prediction model. ISSA has the advantages of high convergence accuracy, fast optimization speed, and good stability. In this paper, an ISSA-SVR prediction model is constructed by using ISSA for parameter search optimization of the SVR prediction model, which means the combination of penalty factor C and kernel function parameter *g* is optimized and its flow chart is shown in Figure 4.

## 5. Water Quality Prediction Experiment

### 5.1. Water Quality Data Collection

According to the water quality standard for aquaculture, five kinds of parameters, such as temperature, pH value, dissolved oxygen, salt content, and ammonia nitrogen content, are usually used to reflect the quality of water. In this paper, the water quality prediction of swimming crab aquaculture is taken as the application object and these five kinds of water quality parameters are selected as the monitoring objects to predict the overall quality of water by time series. The water quality is divided into I, II, III, IV, and V categories from good to poor. The detailed indicators are shown in Table 13.

**Figure 5:**
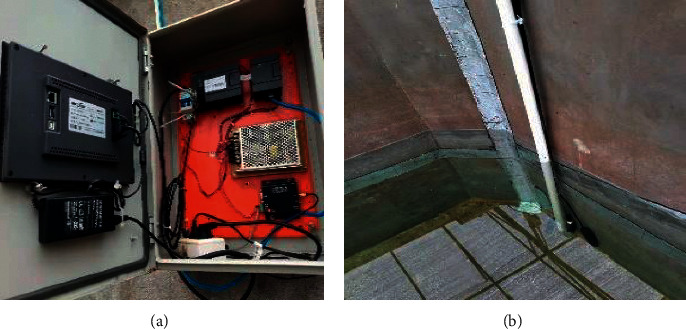
Field acquisition equipment. (a) Data-saving equipment. (b) Data collection equipment.

In this paper, a distributed water quality monitoring platform is built in an aquaculture farm in Xiangshan, Ningbo, China. The high-precision cluster water quality sensor is used to collect water quality data, and the collected water quality data are stored in the SQL Server database of PC. The field equipment diagram is shown in Figure 5.

In this experiment, 3000 groups of data were obtained continuously from the database according to the time series. Each group of data includes five kinds of parameters: temperature, pH value, salinity, ammonia nitrogen, and dissolved oxygen. 210 groups of abnormal data were eliminated. The remaining 2790 groups of effective data were divided into two groups, of which 2290 groups were used as the training set samples of the new algorithm and the remaining 500 groups were used as the test set samples.

### 5.2. Performance Metrics

Two performance measures (PMs) including the mean squared error (MSE) and the coefficient of determination (*R*^2^) are used to evaluate the proposed predictive models [10]. These PMs were calculated as follows:

(20) $$\begin{array}{c} \text{MSE}=\frac{1}{n}\sum_{i=1}^{n}{\left(y_{i}-{\hat{y}}_{i}\right)}^{2}, \end{array}$$

where $\hat{y}$ denotes the output values, *y* denotes the real values, and *n* is the total number of items.

(21) $$\begin{array}{c} R^{2}=1-\frac{\sum_{i=1}^{n}{\left(y_{i}-{\hat{y}}_{i}\right)}^{2}}{\sum_{i=1}^{n}{\left(y_{i}-\bar{y}\right)}^{2}}, \end{array}$$

where $\bar{y}$ is the mean value of the *y* values.

### 5.3. Results and Analysis

To compare the performance of the ISSA-SVR prediction model with other prediction models, BP neural network, SVR, SSA-SVR, TLBO-SVR, SOA-SVR, GWO-SVR, PSO-SVR, and WOA-SVR models are established in the same way. Then, the same data set is used for water quality grade prediction. Finally, MSE, *R*^2^, and running time are compared, that is, the prediction accuracy and convergence speed are compared to evaluate the performance of the prediction model.

This test is carried out by MATLAB 2018b software. Before the test, the data set is normalized by [0,1]. In the test, the range of penalty factor C and kernel function parameter *g* is [0.1, 100], the population size is 30, the maximum number of iterations is 100, the fitness function is the mean square error MSE of the distance between the training sample and the optimal hyperplane, and the minimum MSE is the optimal value. The unified parameter population size is set to 30, and the maximum number of iterations is set to 100. In the BP neural network, there are seven single hidden layer nodes, the transfer function is Tansig, the training function is Trainlm, and the SVR kernel function is set as RBF kernel function. The relevant parameters of other algorithms are shown in Table 11.

**Figure 6:**
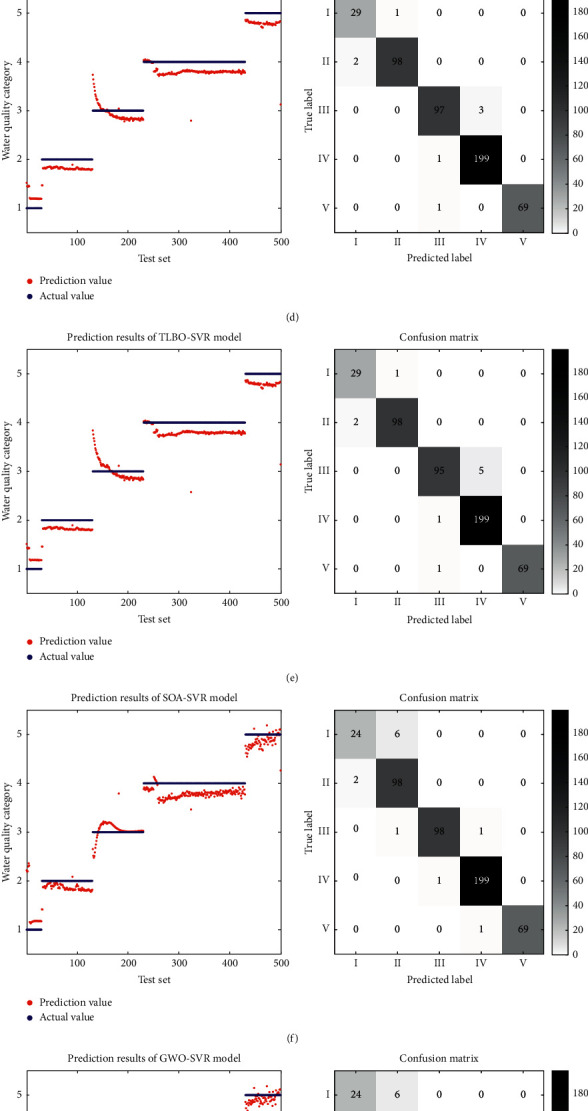
Prediction results and confusion matrix of nine water quality regression models. (a) BP neural network water quality regression prediction results and confusion matrix. (b) SVR water quality regression prediction results and confusion matrix. (c) ISSA-SVR water quality regression prediction results and confusion matrix. (d) SSA-SVR water quality regression prediction results and confusion matrix. (e) TLBO-SVR water quality regression prediction results and confusion matrix. (f) SOA-SVR water quality regression prediction results and confusion matrix. (g) GWO-SVR water quality regression prediction results and confusion matrix. (h) PSO-SVR water quality regression prediction results and confusion matrix. (i) WOA-SVR water quality regression prediction results and confusion matrix.

To compare the predictive performance of the models used in this study (BP neural network, SVR, ISSA-SVR, SSA-SVR, TLBO-SVR, SOA-SVR, GWO-SVR, PSO-SVR, and WOA-SVR models), their metric indices (MSE and *R*^2^) and running time obtained during the testing phase are presented in Table 14. The test results and confusion matrix are shown in Figure 6.

As can be seen in Table 14, the ISSA-SVR model provides better performance (MSE=0.013 and *R*^2^=0.980) than the BP neural network and the conventional SVR model, where especially the MSE is reduced by 90.07% and 79.37%. In terms of running time, the ISSA-SVR model shows increase compared to BP neural network and traditional SVR models, which is the time cost necessary to obtain the optimal parameters using the optimization algorithm. In practical engineering applications, the prediction model can be pretrained and used by simply bringing the optimal parameters into the model, so it is more cost-effective to spend a certain amount of modeling training time to obtain a smaller MSE and a larger *R*^2^.

Comparing the traditional SVR model with ISSA-SVR, SSA-SVR, TLBO-SVR, SOA-SVR, GWO-SVR, PSO-SVR, and WOA-SVR models, it can be found that the hybrid model performs better, indicating that adding the intelligent search algorithm can effectively improve the performance of the SVR model.

Further comparing the performance of the seven hybrid models (ISSA-SVR, SSA-SVR, TLBO-SVR, SOA-SVR, GWO-SVR, PSO-SVR, and WOA-SVR), it can be found that the ISSA-SVR model not only has better performance (MSE=0.013 and *R*^2^=0.980) but also has less running time (time= 80.98). When the ISSA-SVR model is compared with SSA-SVR, TLBO-SVR, SOA-SVR, GWO-SVR, PSO-SVR, and WOA-SVR models, MSE is reduced by 75%, 76.36%, 77.97%, 76.79%, 76.79%, and 76.79%, *R*^2^ is increased by 76.79%, 7.57%, 2.40%, 2.73%, 2.51%, 3.05%, and 2.51%, and running time is reduced by 5.79%, 71.44%, 23.18%, 2.64%, 31.54%, and 2.83%. It shows that ISSA has the best effect on the SVR model parameter search (optimal parameters *C*=0.296629 and  *g*=0.296661).

In particular, when comparing the ISSA-SVR model and SSA-SVR model separately, it is easy to see that the ISSA-SVR model has improved performance and running time, indicating that the introduction of Skew-Tent mapping initialization strategy and adaptive elimination mechanism in the ISSA-SVR model can improve the global searchability and generalization ability of the algorithm, further proving that the proposed ISSA in this paper has better optimization effect compared with the traditional SSA.

In order to further explore the performance of the water quality prediction model, Figure 5 shows the prediction results and confusion matrix of water quality category prediction using the above models, where the darker the color of the confusion matrix represents the more the data predicted correctly. Based on the prediction results in Figure 4, it is clear that the prediction results of the ISSA-SVR model are usually closer to the true value and more accurate than those of other models. From the confusion matrix, we can see that the prediction accuracy of the ISSA-SVR model reaches 99.2%, which is 4.86% higher than that of the SVR model, 0.81% higher than that of the SSA-SVR model, and 6.90% higher than that of the BP model which has the worst prediction effect, and it is the only model which is completely accurate in predicting the water quality of Class I. The prediction effect is obviously better than that of the other models.

Through the previous analysis, it can be fully explained that ISSA-SVR model has better performance than BP neural network, traditional SVR model, and other hybrid models, which can predict the water quality category more accurately and can provide better technical support for water quality management.

## 6. Conclusion

This paper mainly improves the sparrow search algorithm and applies the improved sparrow search algorithm to the parameter optimization of the SVR model, optimizes the combination of penalty factor C and kernel function *g*, and constructs an ISSA-SVR water quality regression prediction model based on the actual aquaculture water quality data:

1. Aiming at the defects of the standard sparrow search algorithm, one solution is to introduce Skew-Tent map initialization strategy to increase the diversity of the initial population and the second solution is to integrate the adaptive elimination mechanism. With the iterative change of the population, the individuals with lower fitness are eliminated adaptively and new individuals are added to the population by using prior knowledge. The experimental results show that the global optimization ability of the improved SSA is enhanced and it can effectively avoid falling into local optimization. The overall convergence accuracy and speed of the improved SSA are significantly better than those of the comparison algorithms, and the algorithm is relatively more stable and robust.
2. An ISSA-SVR water quality prediction model was established. Based on the actual aquaculture water quality data, the parameters C and *g* of the SVR model were optimized with ISSA and the ISSA-SVR water quality prediction model was established. The test results show that the prediction accuracy of the ISSA-SVR model is better than that of the BP neural network, SVR, SSA-SVR, TLBO-SVR, SOA-SVR, GWO-SVR, PSO-SVR, and WOA-SVR model; MSE is 0.013 and *R*^2^ is 0.980, which is close to the actual water quality level and meets the actual needs. Although the running time of BP neural network and SVR models is shorter than that of the ISSA-SVR model, in practical engineering applications, the training models are pretrained and the optimal parameters need to be brought into the model only when used, so it is more cost-effective to spend some modeling training time to obtain smaller MSE and higher *R*^2^. For future research, we can try to analyze the minimum number of iterations to obtain an equivalent training effect, resulting in less training time.
3. In this study, only five water quality classification impact factors were selected from the experimental data and the data correlation between the impact factors was not considered. But, in fact, the factors that affect the water quality judgment are multiple and complex. For the subsequent study with a large number of impact factors, correlation analysis of the factors is needed to select the most important factors that affect water quality and avoid unnecessary calculations. At the same time, the experimental data of this study came from the actual aquaculture plant, and the judging criteria of water quality classification for different farming objects in the plant are different. Future research can try to build a water quality prediction model that meets the water quality requirements of multiple farming objects at the same time.

## Figures and Tables

**Algorithm 1:**
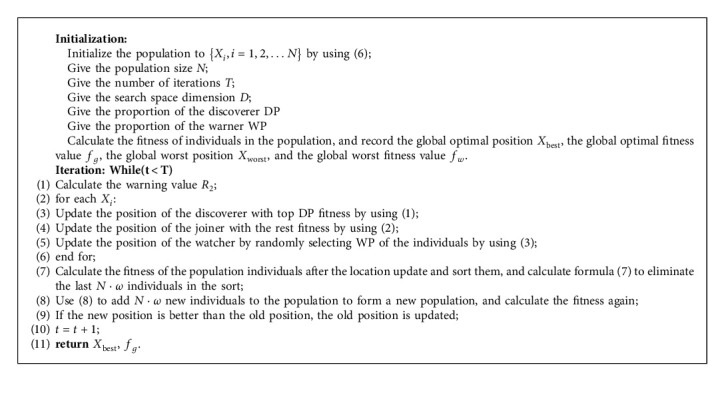
Improved sparrow search algorithm.

**Table 1:** Control factors and their levels of Taguchi experimental design.

Factor	Level		
	1	2	3
The proportion of the discoverer (A)	10%	15%	20%
The proportion of the warner (B)	10%	15%	20%
Population size (C)	10	30	50

**Table 2:** The L9 orthogonal table with response values and SN ratio for each optimization objective.

No.	A (%)	B (%)	C	GP	V	$\bar{t}$	SN (GP)	SN (V)	SN ($\bar{t}$)
1	10	10	10	0.420	0.203	0.157	−8.876	13.652	16.085
2	10	15	50	0.400	0.202	0.156	−11.299	13.591	16.126
3	10	20	30	0.400	0.205	**0.156**	−10.958	13.495	**16.149**
4	15	10	50	0.385	0.207	0.157	−11.448	13.436	16.087
5	15	15	30	0.405	0.206	0.160	−11.007	13.415	15.891
6	15	20	10	0.370	0.209	0.161	−13.237	13.144	15.852
7	20	10	30	**0.475**	**0.182**	0.162	**−7.982**	**14.366**	15.829
8	20	15	10	0.360	0.221	0.161	−10.295	12.773	15.842
9	20	20	50	0.360	0.223	0.161	−11.658	12.737	15.888

The bold values demonstrate the optimal response value and the highest S/N for each optimisation objective.

**Table 3:** Average SN ratio for GP at each factor and level.

Factor	Level 1	Level 2	Level 3	Max-min	Rank
A	−10.378	−11.898	**−9.978**	1.919	2
B	**−9.435**	−10.867	−11.951	**2.516**	1
C	−10.803	**−10.349**	−11.479	1.130	3

The bold values demonstrate the optimal response value and the highest S/N for each optimisation objective.

**Table 4:** Average SN ratio for *V* at each factor and level.

Factor	Level 1	Level 2	Level 3	Max-min	Rank
A	**13.580**	13.332	13.292	0.287	3
B	**13.818**	13.260	13.126	**0.693**	1
C	13.190	**13.678**	13.164	0.514	2

The bold values demonstrate the optimal response value and the highest S/N for each optimisation objective.

**Table 5:** Average SN ratio for $\bar{t}$ at each factor and level.

Factor	Level 1	Level 2	Level 3	Max-min	Rank
A	**16.120**	15.944	15.853	**0.194**	1
B	**16.000**	15.953	15.963	0.047	3
C	15.926	15.989	**16.007**	0.081	2

The bold values demonstrate the optimal response value and the highest S/N for each optimisation objective.

**Table 6:** Grey relational coefficient with their grade and rank.

No.	Grey relational coefficient			Grey relational grade	Rank
	GP	*V*	$\bar{t}$		
1	0.511	0.733	0.980	0.742	2
2	0.434	0.742	0.993	0.723	4
3	0.434	0.712	1	0.715	5
4	0.390	0.699	0.981	0.69	6
5	0.451	0.707	0.925	0.694	3
6	0.354	0.679	0.914	0.649	7
7	1	1	0.908	**0.969**	1
8	0.333	0.598	0.912	0.614	8
9	0.333	0.584	0.924	0.614	9

The bold values demonstrate the optimal response value and the highest S/N for each optimisation objective.

**Table 7:** Average grey relational grade at each factor and level.

Factor	Level 1	Level 2	Level 3	Max-min	Rank
A	0.538	0.482	**0.607**	0.125	3
B	**0.683**	0.486	0.457	**0.226**	1
C	0.478	**0.628**	0.466	0.162	2

The bold values demonstrate the optimal response value and the highest S/N for each optimisation objective.

**Table 8:** Description of unimodal benchmark functions.

Function	Dimension	Range	The optimal value
*F*_1_(*x*)=∑_*i*=1_^*n*^*x*_*i*_^2^	30	[−100, 100]	0
*F*_2_(*x*)=∑_*i*=1_^*n*^|*x*_*i*_|+∏_*i*=1_^*n*^|*x*_*i*_|	30	[−10, 10]	0
*F*_3_(*x*)=∑_*i*=1_^*n*^(∑_*j*=1_^*i*^*x*_*j*_)^2^	30	[−100, 100]	0
*F*_4_(*x*)=max_*i*_{|*x*_*i*_|, 1 ≤ *i* ≤ *n*}	30	[−100, 100]	0
*F*_5_(*x*)=∑_*i*=1_^*n*−1^[100(*x*_*i*+1_ − *x*_*i*_^2^)^2^+(*x*_*i*_ − 1)^2^]	30	[−30, 30]	0
*F*_6_(*x*)=∑_*i*=1_^*n*^([*x*_*i*_+0.5])^2^	30	[−100, 100]	0
*F*_7_(*x*)=∑_*i*=1_^*n*^*ix*_*i*_^4^+random(0,1)	30	[−1.28, 1.28]	0

**Table 9:** Description of multimodal benchmark functions.

Function	Dimension	Range	The optimal value
$F_{8}\left(x\right)=\sum_{i=1}^{n}-x_{i}\sin\left(\sqrt{\left|x_{i}\right|}\right)$	30	[−500, 500]	−418.9829 × 30
*F*_9_(*x*)=∑_*i*=1_^*n*^[*x*_*i*_^2^ − 10 cos(2*πx*_*i*_)+10]	30	[−5.12, 5.12]	0
$F_{10}\left(x\right)=-20\;\exp\left(-0.2\sqrt{1/n\sum_{i=1}^{n}x_{i}^{2}}\right)-\exp\left(1/n\sum_{i=1}^{n}\cos\left(2\pi x_{i}\right)\right)+20+e$	30	[−32, 32]	0
$F_{11}\left(x\right)=\frac{1}{4000}\sum_{i=1}^{n}x_{i}^{2}-\prod_{i=1}^{n}\cos\left(x_{i}/\sqrt{i}\right)+1$	30	[−600, 600]	0
$\begin{array}{c} F_{12}\left(x\right)=\pi /n\left\{10\text{sin}\left(\pi y_{1}\right)+\sum_{i=1}^{n-1}{\left(y_{i}-1\right)}^{2}\left[1+10{\text{sin}}^{2}\left(\pi y_{\left(i+1\right)}\right)\right]+{\left(y_{n}-1\right)}^{2}\right\}+\sum_{i=1}^{n}u\left(\pi x_{i},10,100,4\right)\\ y_{i}=1+\left(x_{i}+1\right)/4u\left(\pi x_{i},a,k,m\right)=\left\{\begin{array}{c} k{\left(\pi x_{i}-a\right)}^{m}x_{i}>a\\ 0-a<x_{i}<a\\ k{\left(-x_{i}-a\right)}^{m}x_{i}<-a \end{array}\right. \end{array}$	30	[−50, 50]	0
*F*_13_(*x*)=0.1{sin^2^(*π*3*πx*_1_)+∑_*i*=1_^*n*−1^(*x*_*i*_ − 1)^2^[1+sin^2^(3*πx*_*i*_+1)]+(*x*_*n*_ − 1)^2^[1+sin^2^(*π*2*πx*_*n*_)]}+∑_*i*=1_^*n*^*u*(*πx*_*i*_, 5,100,4)	30	[−50, 50]	0

**Table 10:** Description of fixed-dimension multimodal benchmark functions.

Function	Dimension	Range	The optimal value
*F*_14_(*x*)=∑_*i*=1_^11^*a*_*i*_−*x*_1_(*b*_*i*_^2^+*b*_*i*_*x*_2_)/*b*_*i*_^2^+*b*_*i*_*x*_3_+*x*_4_^2^	4	[−5, 5]	0.00030
*F*_15_(*x*)=(*x*_2_ − 5.1/4*π*^2^*x*_1_^2^+5/*πx*_1_ − 6)^2^+10(1 − 1/8*π*)cos *x*_1_+10	2	[−5, 5]	0.398
*F*_16_(*x*)=−∑_*i*=1_^4^*c*_*i*_exp(−∑_*j*=1_^6^*a*_*ij*_(*x*_*j*_ − *p*_*ij*_)^2^)	6	[0, 1]	−3.86
*F*_17_(*x*)=−∑_*i*=1_^5^[(*X* − *a*_*i*_)(*X* − *a*_*i*_)^*T*^+*c*_*i*_]^−1^	4	[0, 10]	−10 .1532

**Table 11:** Experimental parameter settings.

Algorithm	Parameter
ISSA	The discoverer accounts for 20%; the warner accounts for 10%
SSA	The discoverer accounts for 20%; the warner accounts for 10%
TLBO	No specific parameters are required
SOA	*a* decreases linearly from [2, 0]; *f*_*c*_=2
GWO	*a* decreases linearly from [2, 0]
PSO	*w*=6, *w*_dawp_=0.9, *C*_1_=1.5, and *C*_2_=2
WOA	*a* decreases linearly from [2, 0]; *b*=1

**Table 12:** Comparison of optimization results obtained for the unimodal, multimodal, and fixed-dimension multimodal benchmark functions.

Function	Statistics	ISSA	SSA	TLBO	SOA	GWO	PSO	WOA

F_1_(*x*)	Best	**0**	**0**	4.3662*e* − 129	8.96714*e* − 10	3.9375*e* − 29	2.3205*e* − 05	7.2814*e* − 85
	Average	**1.9339e** − **75**	3.5155*e* − 71	8.606*e* − 65	1.1142	1.8036*e* − 27	0.00016112	1.6301*e* − 62
	STD	**8.6488e** − **75**	1.4461*e* − 70	2.5987*e* − 64	2.5889	3.3361*e* − 27	0.00017167	6.2939e − 62

F_2_(*x*)	Best	**2.5384e** − **301**	9.932*e* − 244	4.3058*e* − 65	0.00030389	2.6648*e* − 17	0.0030429	1.643*e* − 57
	Average	**7.2433e** − **37**	7.8935*e* − 37	2.658*e* − 33	0.051054	1.2203*e* − 16	0.031152	1.4748*e* − 21
	STD	**3.041e** − **36**	3.5301*e* − 36	8.2999*e* − 33	0.095959	8.6352*e* − 17	0.024356	3.7466*e* − 21

F_3_(*x*)	Best	**0**	**0**	3.055*e* − 129	14076.3527	8.8281*e* − 08	31.28514	8423.09741
	Average	**2.6334e** − **54**	1.8427*e* − 50	1.8078*e* − 44	36379.9353	0.00014427	79.0074	48012.7909
	STD	**1.1777e** − **54**	7.6879*e* − 50	7.6837*e* − 44	11258.5471	0.00058953	30.3177	17635.4437

F_4_(*x*)	Best	**0**	9.7701*e* − 178	8.4257*e* − 63	0.101272	4.269*e* − 08	0.709	2.04388
	Average	2.6512*e* − 42	1.4467*e* − 38	**1.7574e** − **61**	21.3948	9.1125*e* − 07	1.0452	45.1839
	STD	1.1851*e* − 41	6.4618*e* − 38	**1.6742e** − **61**	16.6063	1.0324*e* − 06	0.21658	26.8389

F_5_(*x*)	Best	**3.8905e** − **10**	8.3254*e* − 08	28.8308	28.7010059	25.2907	27.43913	27.5618
	Average	**5.7835e** − **05**	0.00013906	28.9176	4496.0152	27.0187	99.6455	28.0048
	STD	**0.00017638**	0.000274	0.039083	16623.6163	0.75214	64.7196	0.36965

F_6_(*x*)	Best	**1.1506e** − **13**	1.1409*e* − 10	2.3166	0.6211821	7.8096*e* − 05	1.5931*e* − 06	0.12428
	Average	**2.7962e** − **09**	7.9257*e* − 09	5.4424	32.423	0.86889	0.00014457	0.389
	STD	**3.4276e** − **09**	1.1242*e* − 08	0.96486	128.9457	0.33212	0.0001316	0.21333

F_7_(*x*)	Best	**1.5716e** − **05**	8.7234*e* − 05	0.012761	0.0011214	0.00045703	0.070948	0.000101
	Average	**0.00037234**	0.0006554	0.051421	0.055712	0.002143	0.18752	0.0034594
	STD	**0.00035557**	0.00059506	0.03161	0.086087	0.0011226	0.07439	0.0038655

F_8_(*x*)	Best	**−12569.4866**	−8994.1369	−5772.8168	−11292.7044	−8032.753	−6054.6416	−12567.6499
	Average	**−10213.0471**	−7863.8947	−4911.1516	−10173.4353	−5926.5447	−4599.8435	−9785.6132
	STD	2970.6002	755.2518	**472.0635**	690.6512	899.251	1116.2956	1601.3101

F_9_(*x*)	Best	**0**	**0**	**0**	4.695037*e* − 07	5.68434*e* − 14	28.9705	**0**
	Average	**0**	**0**	**0**	22.3091	3.7179	61.0113	**0**
	STD	**0**	**0**	**0**	43.511	5.297	13.8801	**0**

F_10_(*x*)	Best	**8.8818e** − **16**	**8.8818e** − **16**	4.4409*e* − 15	0.0018817	7.5495*e* − 14	0.0027606	**8.8818e** − **16**
	Average	**8.8818e** − **16**	**8.8818e** − **16**	4.2633*e* − 15	0.15716	9.5035*e* − 14	0.28322	3.5527*e* − 15
	STD	**0**	**0**	7.9441*e* − 16	0.31464	1.3368*e* − 14	0.49281	1.9544*e* − 15

F_11_(*x*)	Best	**0**	**0**	**0**	0.00023624	**0**	7.9754*e* − 07	**0**
	Average	**0**	**0**	**0**	0.40922	0.0063389	0.0093781	0.010225
	STD	**0**	**0**	**0**	0.47094	0.010786	0.01022	0.045728

F_12_(*x*)	Best	**2.7334e** − **13**	4.0027*e* − 13	0.15793	0.0725647292	0.017097	5.6583*e* − 08	0.0035851
	Average	**9.8583e** − **09**	3.6537*e* − 10	0.62116	4298.3255	0.036334	0.010369	0.017069
	STD	**1.6303e** − **09**	7.3888*e* − 10	0.23345	18301.5024	0.017898	0.031909	0.0089687

F_13_(*x*)	Best	**2.6708e** − **12**	7.6948*e* − 11	2.0234	0.196980813	0.3364	1.7361*e* − 06	0.11787
	Average	4.2144*e* − 08	**3.4782e** − **09**	2.6905	2140.8307	0.63224	0.0034512	0.47959
	STD	5.9292*e* − 08	**5.0163e** − **09**	0.30738	6462.5832	0.25999	0.0050935	0.24911

F_14_(*x*)	Best	**0.00030749**	**0.00030749**	**0.00030749**	0.0007919	0.00030758	0.00066519	0.00031293
	Average	0.00030823	**0.00030755**	0.0013561	0.0059898	0.0043713	0.00090965	0.00056477
	STD	**1.0008e** − **07**	1.1279*e* − 07	0.0044785	0.0054051	0.0082039	0.00011385	0.00025792

F_15_(*x*)	Best	**0.39789**	**0.39789**	**0.39789**	**0.39789**	**0.39789**	**0.39789**	**0.39789**
	Average	**0.39789**	**0.39789**	**0.39789**	0.40883	**0.39789**	**0.39789**	**0.39789**
	STD	**0**	**0**	**0**	0.013943	7.4343*e* − 07	**0**	5.5277*e* − 06

F_16_(*x*)	Best	**−3.322**	**−3.322**	−3.3218	−3.2867	**−3.322**	**−3.322**	−3.3213
	Average	**−3.2863**	−3.2507	−3.2505	−3.0138	−3.2858	−3.2685	−3.1963
	STD	**0.055899**	0.059759	0.067634	0.20914	0.066084	0.060685	0.22532

F_17_(*x*)	Best	**−10.1532**	**−10.1532**	**−10.1532**	−9.5161	−10.1525	**−10.1532**	−10.1524
	Average	**−9.7429**	−8.8786	−8.8787	−5.9867	−9.6464	−8.3957	−8.2817
	STD	**1.469**	2.2634	2.2648	1.7488	1.5551	2.8279	3.0489

The bold values demonstrate the optimal response value and the highest S/N for each optimisation objective.

**Table 13:** Water quality index of swimming crab culture (mg/L).

Classification	I	II	III	IV	V
Temperature (°C)	25	20/30	15/35	10/40	5/45
pH value	8.0	7.5/8.5	7.0/9.0	6.5/9.5	6.0/10.0
Salinity	28	25/30	20/35	15/40	10/45
Ammonia nitrogen	0	0.5	1.0	1.5	2.0
Dissolved oxygen	≥10.0	≥6.0	≥5.0	≥3.0	≥2.0

**Table 14:** Running time, MSE, and *R*^2^ of nine prediction models.

Index	MSE	*R* ^2^	Time (s)
BP neural network	0.131	0.973	0.45
SVR	0.063	0.930	**0.32**
ISSA-SVR	**0.013**	**0.980**	80.98
SSA-SVR	0.052	0.911	85.99
TLBO-SVR	0.055	0.957	283.52
SOA-SVR	0.059	0.954	105.42
GWO-SVR	0.056	0.956	83.18
PSO-SVR	0.056	0.951	118.28
WOA-SVR	0.056	0.956	83.34

The bold values demonstrate the optimal response value and the highest S/N for each optimisation objective.

## Data Availability

The data used to support the findings of this study are available from the corresponding author upon request.
